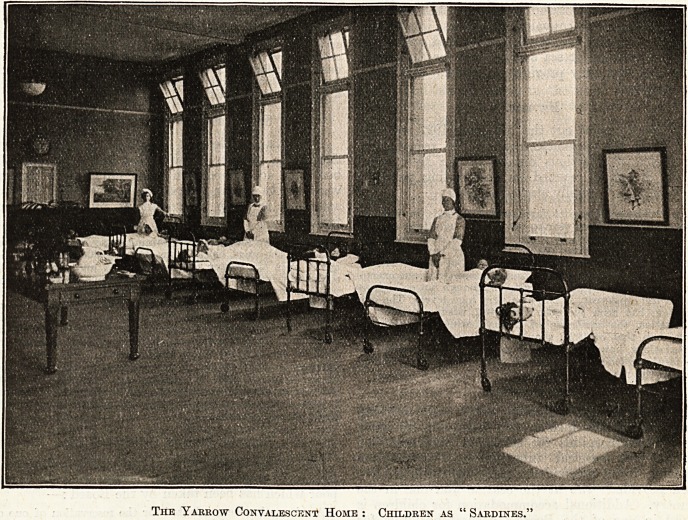# The Head-To-Foot Way

**Published:** 1923-09

**Authors:** Alfred Yarrow


### THE HEAD-TO-FOOT WAY.

### NEW DORMITORY METHOD.

By SIR ALFRED YARROW, F.R.S.

[Sir Alfred Yarrow's rearrangement of the beds in his Convalescent Home for Children at Broadstairs, as a means of reducing the risk of infection, has attracted a good deal of attention. In the following article Sir Alfred describes the innovation, and the reasons for it.]

IT will be seen from the accompanying photograph that the heads of the children are placed alternately next to the wall and away from the wall. If they are all next to the wall the distance between the faces of the children is very much less than when they are arranged sardine fashion, and the risk of infection is more or less in proportion to the square of the distance. At any rate, it is common sense and self-evident that the nearer the infected child is to its uninfected neighbour, the more risk there is of the uninfected child catching the complaint. Sir James Crichton Browne has drawn my attention to the fact that with children suffering from surgical trouble it is very bad for them to twist their bodies round to talk to one another, which they do when the heads are all next to the wall, while when the beds are arranged sardine fashion they would face one another and would not need to distort their bodies when they want to talk. The risk of infection would be less among men than among children by adopting the sardine fashion, because the heads would be further away than they would be in the case of the children.

It may be said that the light shines in the faces of some of the children. The objection to that can be overcome by curtains; and in dormitories where the windows are on both sides that contention would not hold good. The only other objection I have heard is that the new method does away with the symmetry of the dormitory, but I think our eyes would soon be educated to the new arrangement.

THE YARROW CONVALESCENT HOME: CHILDREN AS "SARDINES."

I may say that this method of arranging the beds is no suggestion of mine. My attention was first drawn to the matter by an article in the "Times." I think all institutions and schools ought to arrange the beds in this way, with a view to reducing the spreading of illnesses of an infectious character, which is one of their greatest difficulties.

---

### Travelling Scholarship Prize Award.

The winner of the Scholarship Prize Award, in connection with the Sims Woodhead series of constructive Educational Health Lectures recently given by the League at the headquarters of the National Union of Teachers, is Miss Alice Gloyn. Miss Gloyn, who has been in charge of the Camberwell Day Sanatorium since 1917, working under the London County Council in conjunction with the Tuberculosis Officer for Camberwell, has chosen to attend Dr. A. Rollier's course of lectures on heliotherapy at Leysin in Switzerland. She will also visit the Graucher and other institutions in France dealing with tuberculosis in children.

## Figures and Tables

**Figure f1:**